# Healthcare Avoidance before and during the COVID-19 Pandemic among Australian Youth: A Longitudinal Study

**DOI:** 10.3390/healthcare10071261

**Published:** 2022-07-06

**Authors:** Md Irteja Islam, Joseph Freeman, Verity Chadwick, Alexandra Martiniuk

**Affiliations:** 1 Sydney School of Public Health, Faculty of Medicine and Health, The University of Sydney, Edward Ford Building, A27 Fisher Road, Sydney, NSW 2006, Australia; m.i.islam@sydney.edu.au (M.I.I.); jfre7317@uni.sydney.edu.au (J.F.); 2 Centre for Health Research and School of Sciences, The University of Southern Queensland, West Street, Toowoomba, QLD 4350, Australia; 3 Royal North Shore Hospital, Reserve Rd., St. Leonard’s, Sydney, NSW 2065, Australia; veritychadwickjackman@gmail.com; 4 Office of the Chief Scientist, The George Institute for Global Health, Level 5/1 King Street, Newtown, Sydney, NSW 2042, Australia; 5 Dalla Lana School of Public Health, The University of Toronto, 155 College St. Room 500, Toronto, ON M5T 3M7, Canada

**Keywords:** COVID-19 pandemic, coronavirus, service access, healthcare avoidance, perceived need, adolescents, youth, young adult, Australia

## Abstract

Background: Access to healthcare for young people is essential to ensure they can build a foundation for a healthy life. However, during the COVID-19 pandemic, many people avoided seeking healthcare, adversely affecting population health. We investigated the factors associated with the avoidance of healthcare for Australian young people when they reported that they needed healthcare. We were able to compare healthcare avoidance during the COVID-19 pandemic with healthcare avoidance prior to COVID-19. Methods: We used two recent data collection waves from the Longitudinal Study of Australian Children (LSAC)—Wave 9C1 during the COVID-19 pandemic in 2020, and Wave 8 data which were collected in 2018. The primary outcome of this study revealed the avoidance of healthcare among those who perceived the need for care. Bivariate analyses and multiple logistic regression models were employed to identify the factors associated with the avoidance of healthcare during the COVID-19 and pre-COVID-19 periods. Results: In the sample of 1110 young people, 39.6% avoided healthcare during the first year of the COVID-19 pandemic even though they perceived that they had a health problem that required healthcare. This healthcare avoidance was similar to the healthcare avoidance in the pre-COVID-19 pandemic period (41.4%). The factors most strongly associated with healthcare avoidance during the COVID-19 pandemic were female gender, an ongoing medical condition, and moderately high psychological distress. In comparison, prior to the pandemic, the factor associated with healthcare avoidance was only psychological distress. The most common reason for not seeking healthcare was thinking that the problem would spontaneously resolve itself (55.9% during COVID-19 vs. 35.7% pre-COVID-19 pandemic). Conclusions: A large proportion of youths avoided healthcare when they felt they needed to seek care, both during and before the COVID-19 pandemic.

## 1. Introduction

Access to healthcare is key to maintaining health and optimising disease management. However, healthcare access has been reduced in both high-income and low-and-middle-income countries during COVID-19, resulting in increased mortality and morbidity [1,2]. Access to paediatric healthcare, in particular, has reduced dramatically in countries including China [3], the USA [4], and Germany [5]. Adolescents and young adults were identified as vulnerable to the direct and indirect effects of COVID-19 due to their reduced access to healthcare [6]. Health systems have been burdened by waves of COVID-19, resulting in decreased healthcare resources to manage COVID-19 and non-COVID-19-related conditions [7]. Access to healthcare has also been impeded by mandated isolation, travel restrictions, lost or reduced income and support, and the perceived risk of COVID-19 to oneself and vulnerable persons [8]. Moreover, large proportions of outpatient and primary healthcare have moved to telehealth since the declaration of COVID-19 as a global pandemic in 2020 [9,10]. This unprecedented and sudden reconstruction of the healthcare system might account for some of the reductions in service access, but it could also be explained by patients’ avoidance of healthcare due to a fear of contracting COVID-19 [11]. It is commonly known that ‘avoidance’ refers to the act of physically and/or mentally moving away from something, and has been described previously in the context of traumatic or threatening situations such as this COVID-19 pandemic [11,12]. Healthcare avoidance is often defined by missing appointments, failing to adhere to therapy, and delaying or avoiding medical care due to cost, time, fear, or a denial of symptoms, among other variables [12,13]. Research conducted among adults found that a lack of the perceived need for healthcare, having comorbid conditions, and living in an area with high COVID-19 prevalence have also led to the avoidance of healthcare in the past two years [13,14,15].

In several countries, regardless of COVID-19 incidence rates, the proportion of individuals who delayed or avoided healthcare access increased [11]. For example, a study reported about 40% of the surveyed sample (n = 4977) avoided healthcare due to COVID-19 when the total number of COVID-19 cases was around 2.5 million in the US [15], while another study conducted in Korea found that more than 70% of their respondents (n = 1000) avoided healthcare in response to the COVID-19 pandemic [13]. A study in the UK compared the first year of the COVID-19 pandemic with the 4 years prior and observed a 38.1% (95% CI 33.9% to 42.3%) reduction in emergency department presentations during the pandemic with no differences observed by sex, age, deprivation, or ethnicity. Hospital admissions in the UK were also lower during the pandemic, with a 23.4% (17.4% to 29.4%) reduction, though less of a reduction was observed for admissions for the 5–17-year-old age group [16].

In Australia, a study of adults during the COVID-19 pandemic in the state of Victoria found that almost one-third of the respondents (n = 1260) either delayed or avoided healthcare due to COVID-19 concerns [14]. Another study of health service use during the pandemic in Victoria, Australia, showed reduced hospital presentations among the young and elderly during the pandemic. However, healthcare access for urgent conditions at the population level remained constant [17]. A study of paediatric health service use in the state of New South Wales (NSW), Australia, found significantly lower attendance in the 2020 lockdown for chronic conditions, acute infections, and injuries [18]. After the lockdown, hospital presentations returned to pre-COVID-19 levels, except for mental health presentations, which remained 30–55% higher than predicted [18].

In Australia, despite using a public/private healthcare system with most hospital care being publicly funded [19,20], the COVID-19 pandemic has affected young people in multiple ways. Australians aged 20–29 have had the highest total proportion of positive cases [21]. Although COVID-19 is less severe in young people on average [6], concerns remain regarding the chance of severe illness, long COVID-19, onward transmission, and foregone non-COVID-19 care [22]. Emergency Department (ED) presentations for young people aged 15–24 dropped by 3.9% points from 2018–2019 to 2019–2020, with the latter period spanning 1 July 2019 to 30 June 2020 [23]. A study comparing five years of Australian ED data before the pandemic and then the pandemic months until February 2021 showed a 38.1% (95% CI 33.9% to 42.3%) reduction in ED presentations during the pandemic with no differences by age, gender, deprivation, or ethnicity [16]. Further investigated hospital admission change during the pandemic compared to the four years prior and found there was a 23.4% (17.4% to 29.4%) reduction in hospital admissions. The study revealed that there were fewer admissions for infection and respiratory conditions, the same number of admissions for other conditions, but more hospital admissions among adolescent females for mental illness [16].

Young people in aggregate require less healthcare for acute and chronic illnesses than older age groups [24]. However, health in adolescence and young adulthood can set a lifelong trajectory [25]. For children with acute problems, access to safe and effective healthcare reduces the risk of complications and interruption to physical and mental development. For children with chronic health conditions, optimising management early, which requires access to healthcare, ensures the best outcomes [26]. Young people’s mental and physical health has been adversely affected during the COVID-19 pandemic, and this is compounded by an avoidance of healthcare [23,27]. Researchers have demonstrated that individuals who avoided healthcare due to a fear of infection experienced an increased severity of their diseases and an increased mortality has been observed in part due to greater barriers to treatment [11]. In Australia, the COVID-19 pandemic worsened three-quarters of young people’s mental health, resulting in greater help-seeking for mental health problems during pandemic periods, whereas help-seeking for other health issues was either similar or reduced [28,29,30,31].

Healthcare avoidance has been reported worldwide [11,13,32]. Contextualising real or perceived healthcare access barriers and identifying factors related to avoiding healthcare can help healthcare systems, researchers, and policymakers find solutions to overcome these barriers [33,34,35]. Recent population-based studies conducted only among adults suggest that aged people, females, individuals with chronic diseases (e.g., palpitations, limb weakness, or chest pain), cost, and geographic locations are attributable to healthcare avoidance during the COVID-19 pandemic [36,37,38]. Prioritising access to safe and desirable healthcare for young people is vital, as without such care they can fall behind in physical, psychosocial, and educational development, having long-lasting effects. To date, no studies in Australia have examined the impact of the COVID-19 pandemic on healthcare access among adolescents who perceived the need for care but avoided it. One existing study has compared global healthcare attendance among children during the COVID-19 pandemic with healthcare attendance before COVID-19, and this was done using a time series analysis of paediatric emergency department presentations providing differences in the rates of presentation before and during the pandemic to one hospital in the UK [16]. To date, the research on this topic has measured healthcare attendance. Purposeful healthcare avoidance is one factor that affects healthcare attendance metrics and has yet not been studied in a longitudinal sample of adolescents before and during the pandemic. 

Therefore, in this study, we sought to: (a) estimate the rate of healthcare avoidance among young people who perceived a need for care during the COVID-19 pandemic compared to before COVID-19 using data from a large longitudinal study, and (b) identify the factors associated with avoiding healthcare, using two of the latest survey datasets (the COVID-19 wave in 2020 and pre-COVID-19 wave in 2018) for a cohort from the Longitudinal Study of Australian Children (LSAC). We anticipated that healthcare avoidance would differ across the two cohort study waves (COVID-19 and pre-COVID-19), and that predictors of healthcare avoidance would be identified among Australian youth who perceived the need for healthcare. 

## 2. Methods

### 2.1. Data Source

We utilized data from the Growing Up in Australia: The Longitudinal Study of Australian Children (LSAC) survey. The LSAC used a multi-stage cluster sampling technique on a complex probability sample to provide credible population estimates. The sampling technique of the LSAC included: (a) stratification—representative postcodes were selected by employing the probability proportion to size method, stratified by state or territory and by capital city statistical division vs. rest of state to guarantee geographically proportionate samples across urban and rural areas; (b) clustering—children were randomly selected from a selection of 311 postcodes, around 40 and 20 children per postcode in the large and small states, respectively; and (c) weighting—LSAC included child population and sample weights in the dataset to offset potential non-response bias and to produce population estimates [39,40]. The LSAC has been collecting data biennially since 2004 from two cohorts: a younger B-cohort (aged 0–1 year at baseline) and an older K-cohort (aged 4–5 at baseline). In total, 10,090 children were recruited during the baseline survey (termed Wave 1 by the LSAC) in 2004, and in the following waves, data were gathered from the same participants as they aged from 2004 to 2020. The details of the LSAC study design and data collection procedures are described elsewhere [40,41].

In this study, we used two recent LSAC waves—Wave 9C1 (i.e., data collection conducted during the COVID-19 pandemic between October and December 2020, referred to here as the ‘COVID-19 pandemic’) and Wave 8 (i.e., data collected in 2018 before the COVID-19 pandemic, referred to here as ‘before the COVID-19 pandemic’ or ‘pre-COVID-19’) to include a sample of 1110 respondents only from the older K-cohort (who were aged 20–21 years during the COVID-19 wave, and were aged 18–19 years in the pre-COVID-19 wave) of the LSAC database [41]. We used K-cohort as only this cohort responded to the same questions regarding healthcare avoidance in both the 2018 and 2020 data waves.

**Figure 1:**
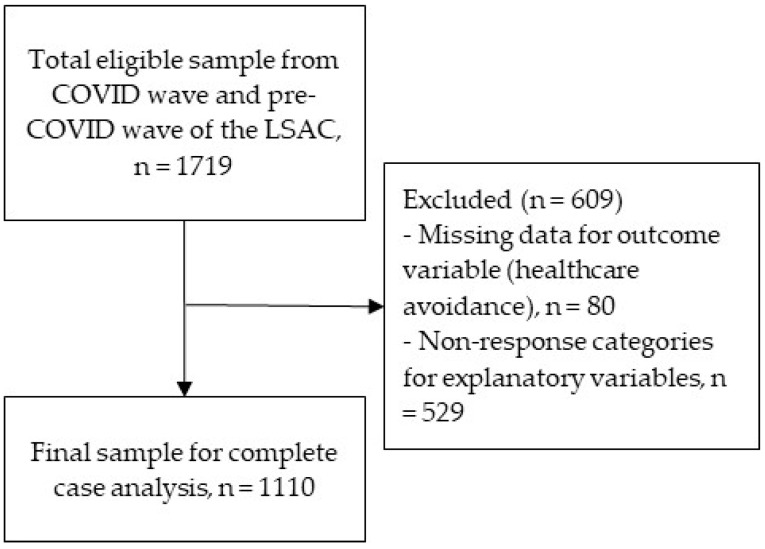
Flow diagram of sample selection.

Figure 1 shows the flow diagram that was used for selecting the final analytical sample. Among the older K-cohort, we found that 1719 participated in both waves (matching unique child ID numbers across the COVID-19 wave and pre-COVID-19 wave). Then, we omitted those participants (n = 609) who did not complete the question about the outcome variable (avoidance of healthcare) and exposure variables (e.g., ongoing medical conditions, psychological distress, family cohesion, etc.) questions in full. Finally, we included 1110 participants as our final analytical sample and performed a complete case analysis (CCA) as the LSAC data were missing completely at random [39,42] and missing variables were not associated with outcome variables. We preferred to use the CCA approach as then the analyses would be based on raw data rather than simulated data; therefore, the CCA generally produces unbiased estimations in regression models [43]. Further, while multiple imputations are particularly useful if it is necessary to preserve sample size, in this study, we have a reasonable sample size, so we used CCA to ensure precision and least biased estimates [44].

### 2.2. Measures

According to Andersen’s Behavioural Model of Health Services Utilization [45], several studies have shown that predisposing factors (e.g., age, sex, education, and employment), enabling factors (e.g., remoteness and socioeconomic status), and need for care (e.g., health-related factors—ongoing medical conditions and psychological distress) are potential predictors of individuals’ accessing or avoiding healthcare [46,47]. In this study, we listed the variables in Table 1 that were deemed to be potential determinants of healthcare seeking or avoidance in accordance with previous studies conducted among young people [48,49,50]. In addition, we included variables related to the COVID-19 pandemic including the coronavirus restriction period (CRP) (also known as ‘lockdown’) between March-May 2020, and these were only available from the COVID-19 wave (i.e., LSAC Wave 9C1 data) [41].

### 2.3. Statistical Analysis

Descriptive statistics in terms of frequency (n) and percentages (%) described the characteristics of the sample, distribution of access or avoidance of services among those who perceived the need for healthcare, and the reasons for avoiding healthcare. Bivariate analyses (using Pearson’s Chi-square test) were used to examine the association between independent variables and the outcome variable (avoidance of healthcare). Finally, two different logistic regression models were employed to identify the predictors of healthcare avoidance during the COVID-19 pandemic (Model I—data used from COVID-19 wave) and pre-COVID-19 period (Model II—data from 2018), respectively. We only included the variables in multiple logistic models that were significantly (*p* < 0.05) associated with healthcare avoidance in the bivariate analysis. Regression results were presented in the form of adjusted odds ratios (OR) with 95% confidence intervals (CI). All data were weighted to account for LSAC’s multi-clustered study design and analyses were implemented using the ‘SVY’ package of Stata version 14.1. 

## 3. Results

The characteristics of the samples are detailed in Table 2. A total of 1110 youths were selected for the study, using two LSAC waves—during the COVID-19 pandemic (Wave 9C1) and during the pre-COVID-19 period (Wave 8).

The study population included 651 (58.6%) females with a mean age of 20.63 years (SD = ±0.49). Most of the respondents were born in Australia, nearly 55% of youths were from NSW and Victoria combined, and 76.5% were from major cities. A total of 64% were enrolled in university or tertiary level education and 77.7% were employed. Overall, 71.5% of young people were living with their parents, almost 85% reported strong family cohesion, and nearly 70% of youths were from disadvantaged socioeconomic groups (Quartiles 1–3). Further, 62% had self-reported ongoing medical conditions, and almost two-thirds had moderate to high psychological distress. 

**Figure 2:**
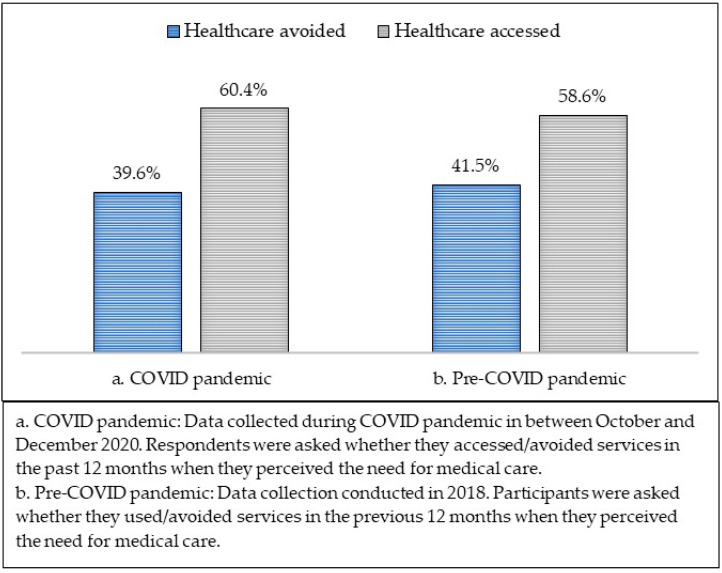
Healthcare access vs. healthcare avoidance during and prior to COVID-19 pandemic.

Figure 2 depicts the distribution of healthcare accessed and avoided (in the past 12 months prior to the survey) in all respondents who perceived the need for care during the COVID-19 pandemic and pre-COVID-19 period.

About 40% of the respondents avoided healthcare in the past 12 months during COVID-19 in 2020, compared to 42% before COVID-19 in 2018. Table 3 provides a detailed breakdown of the reasons for those who avoided healthcare during the COVID-19 pandemic and pre-COVID-19 period. In both periods, most respondents avoided healthcare either because they thought the health problem would be resolved or had already been resolved. In addition, a significant proportion reported an avoidance of healthcare because they were afraid of doctors or visiting healthcare, and this was more pronounced during the COVID-19 pandemic.

The bivariate analysis in Table 4 shows that sex, family cohesion, ongoing medical conditions, and psychological distress were significantly associated with healthcare avoidance during the COVID-19 pandemic. Whereas before COVID-19, besides these variables, living with one’s parents was also found to be significantly associated with whether an adolescent avoided healthcare or not. Regarding COVID-19-related factors during the COVID-19 pandemic, those who reported trouble in life during COVID-19, and who performed more physical activity during the COVID-19 lockdown, were also significantly associated with avoiding healthcare in the bivariate analysis.

The results from the regression models are displayed in Table 5. Model I (using data from the COVID-19 wave) in Table 5 shows that those who had ongoing medical conditions were 1.38 times (95% CI: 1.13–1.70) more likely to avoid healthcare than those who had no ongoing illnesses during the COVID-19 pandemic.

Moreover, moderate (OR 2.06, 95% CI: 1.35–3.18) and high (OR 4.77, 95% CI: 3.57–6.37) rates of psychological distress among youths were associated with a higher likelihood of avoiding healthcare compared to those who reported low psychological distress during COVID-19. Further, Model I show that females were 1.27 times (95% CI: 1.01–1.65) more likely to avoid healthcare than males during the COVID-19 period. Whereas in Model II (using data from before COVID-19 in 2018, Table 5), the variable associated with the avoidance of healthcare included those with moderate to high psychological distress compared to those who reported low/no psychological distress. 

## 4. Discussion

Our study has estimated the rates and factors related to healthcare avoidance among young people during COVID-19 and compared this to the pre-COVID-19 period using longitudinal data. Overall, 39.6% of young Australian respondents avoided healthcare when it was required during the COVID-19 pandemic in 2020. This was a similar proportion to those who avoided healthcare before COVID-19 (41.4%) in 2018. This suggests that the COVID-19 pandemic did not significantly affect young people’s decisions to avoid healthcare even when they perceived the need for care. This may be because Australia was one of the few countries that managed to keep community transmission of COVID-19 very low during 2020, including having periods of no community transmission of COVID-19 between relatively small waves of infection in some parts of the country [29,51]. Moreover, in 2020, the majority of the COVID-19 cases in Australia were in two states: New South Wales and Victoria [29]. Despite this, the Australian State and Territory governments responded swiftly to the COVID-19 waves in 2020 by imposing strict restrictions (e.g., interstate borders were closed, people could only leave their houses for essential items, and so on) to limit the spreading of the virus [52]. Healthcare seeking between waves and in other states of the country may have continued quite similarly to the years before COVID-19 [29,51,53]. Furthermore, similar to other developed countries [54,55,56], Australia has provisioned telehealth services during the COVID-19 pandemic [30], and this might have enabled care seeking to be similar to pre-pandemic [29]. Other researchers have also studied the issue of healthcare avoidance during COVID-19. These have been cross-sectional surveys mostly in adults and have found: 41% of Americans avoided or delayed seeking healthcare during the first year of the COVID-19 pandemic [15], 73% of respondents in South Korea avoided healthcare during the COVID-19 pandemic [13], and 44% in Portugal [11] and 20% in Rotterdam in the Netherlands avoided seeking healthcare during the pandemic [38]. Studies of healthcare utilization have found similar decreases during the COVID-19 pandemic [57].

We found that during the COVID-19 pandemic, females avoided healthcare more than males, which is consistent with previous studies [36,38]. Women generally tend to seek healthcare more than men [58]. Compared to men, women were more likely to be disadvantaged during the COVID-19 pandemic, as they were more likely to lose their jobs and/or work greater hours of unpaid labour, e.g., as caregivers, and were less often recipients of government support [59]. This likely lead to increased stress as well as less time and money to seek healthcare. Furthermore, an American survey found women attended preventive health services less than men during the pandemic and did not present for recommended medical investigations and treatments [60].

Similar to previous studies [37,38], our study found that adolescents and young adults with an ongoing illness were more likely to avoid healthcare than those without any illness. This effect was amplified during the COVID-19 pandemic and could be attributed to a concern of contracting COVID-19, given the greater risk of poorer outcomes for COVID-19 in individuals with comorbidities [61]. Another reason could be the disproportionate side-effects of the COVID-19 pandemic, including changes to travel, isolation requirements, and the economic impact including self and/or family members losing income or changing employment. Furthermore, adolescents and young adults with any illness might have had home environments affected by their own and/or parental stress and mental health issues during the pandemic. Parents and caregivers might have experienced increased home demands and decreased support from outside the home, potentially impairing the ability of youths with disability or illnesses to seek care even though they perceived the need for care [62].

Moreover, our study’s findings indicate that young adults with moderate or high psychological distress were more likely to avoid healthcare during and prior to the COVID-19 pandemic than those who had low/no distress [30]. These findings are supported by a recently conducted population-based study in the Netherlands, which reported a higher level of stress significantly associated with healthcare avoidance [38]. The evidence suggests that healthcare access for youths who perceive the need for care is often complicated by a lack of knowledge and understanding about the process of seeking help, fears of stigmatization, a preference for self-reliance, concerns regarding confidentiality, and a lack of resources including money and the availability of professional help [63,64,65]. Other studies of healthcare avoidance, access, and utilization during COVID-19 have found similar conclusions. Other studies, mostly in adults, have also found healthcare avoidance during COVID-19 to be associated with being older, female, having underlying conditions or disability, having a lower level of education, unemployment, a lower socio-economic status, a reduced trust in government and healthcare response to COVID-19 [11], and having high levels of depression and anxiety [11,13,38,57]. Our study was unique in determining that the proportion of youth who avoided healthcare was similar before COVID-19 as it was during the COVID-19 pandemic.

Our study has limitations. For instance, the study sample was not representative of Australian youths; there was an under-representation of Australians born overseas (5% vs. population 30%) [64] and living in rural or remote areas (23% vs. population 28%) [65]. Furthermore, the data regarding healthcare avoidance, poor health outcomes, and sociodemographic variables (e.g., family cohesion, living with parents, etc.) were self-reported; therefore, they might have been affected by social-desirability bias and recall bias. Another limitation is that this paper describes healthcare avoidance among those who perceived the need for healthcare, but not the actual use of health services. A relative strength is that these data were collected using the same question and in the same way (self-reported) both in the pre-COVID-19 wave and the COVID-19-wave. Moreover, we cannot ascertain whether the individuals’ perceived barriers to healthcare access, or perhaps their healthcare need, was not acute, and whether they sought care later or the problem resolved on its own. In addition, we were not able to include some key variables (e.g., previous history of mental health problems and previous use of healthcare) due to data limitations. Future research may benefit from using objective measures of actual services sought compared to self-reported data as well as more in-depth qualitative methods to increase our understanding of these findings and to further contextualise the reasons for avoiding healthcare, particularly among young adults.

In conclusion, a significant proportion of young adults avoided healthcare when they felt they needed to seek healthcare during and before the COVID-19 pandemic. Whereas COVID-19 did not make a difference in terms of the proportion of youth who avoided healthcare when they felt care was needed. COVID-19 did make a difference in terms of who avoided healthcare. There are similarities as well as differences with respect to who avoided healthcare in 2018 compared to the initial year of the COVID-19 pandemic. In terms of similarities, pre COVID-19 in 2018, as well as during COVID-19 in 2020, youth experiencing moderate or high psychological distress were significantly more likely to report avoiding healthcare when care was perceived to be needed. In terms of differences, being female and having ongoing medical conditions were characteristics that were significantly associated with avoiding healthcare during the first year of the COVID-19 pandemic, but these characteristics did not significantly predict healthcare avoidance before the COVID-19 pandemic. Improved infection control practices in healthcare facilities and the communication of these practices with the public may help to improve healthcare-seeking among people with ongoing medical conditions during pandemics. A better understanding of why women were more likely to avoid healthcare during the COVID-19 pandemic compared to before the COVID-19 pandemic is needed to plan mitigation measures. The most common reason for avoiding healthcare when it was felt to be needed was because the youth thought that the problem would go away. Moreover, during the coronavirus restriction period (the “lockdown”), the most common reason for healthcare avoidance when it was felt to be needed was because the youth did not want to visit a doctor during the lockdown, with the next most common reason being that telehealth was the only appointment option available at the time. These findings highlight the importance of targeted public health education to encourage these young adults to seek healthcare in a timely way, so that their symptoms reduce and consequently their probability of morbidity and/or mortality reduces as well.

## Figures and Tables

**Table 1:** List of variables.

Variables	Description
Outcome variable	
Avoidance of healthcare	The main outcome variable of the study was avoidance of healthcare among those who perceived the need, assessed by asking the cohort “In the last 12 months, has there been any time when you thought you should get medical care, but you didn’t?”. The response categories were ‘Yes’ (coded as 1) and ‘No’ (coded as 0). This is to note that the question was not very sensitive as the LSAC database did not allow us to ascertain how hard or how many times the respondents tried to get access, or how many times they failed to obtain access to services; instead, it provided the list of causes for avoiding the services when the respondents perceived the need.
Exposure variables	
Age	Considered as a continuous variable
Sex	Dichotomized into two categories: ‘Male’ (coded as 0) and ‘Female’ (coded as 1)
Country of birth	Classified as ‘Overseas’ (coded as 0) and ‘Australian’ (coded as 1)
Residential state	Categorized into four: ‘Others’ (coded as 0), ‘New South Wales’ (coded as 1), ‘Victoria’ (coded as 2), and ‘Queensland’ (coded as 3).
Remoteness	According to the Australian Bureau of Statistics (ABS) from the Census of Population and Housing 2016, remoteness areas divide Australia into 5 categories of remoteness based on the relative availability of services—major cities, inner regional, outer regional, remote, and very remote. In this study, we created a binary variable ‘Remoteness’ from the responses. ‘Major cities’ were coded as ‘1′, while ‘inner regional’, ‘outer regional’, ‘remote’, and ‘very remote’ were combined to classify as ‘regional/remote’ (coded as 0).
Education	The education of the participants was dichotomized into two categories: ‘Technical/Others’ (coded as 0) and ‘University/Tertiary’ (coded as 1)
Employment	The employment status of the respondents was dichotomized into two categories: ‘Unemployed’ (coded as 0) and ‘Employed’ (coded as 1).
Living with parents	Dichotomized into two categories: ‘No’ (coded as 0) and ‘Yes’ (coded as 1).
Family cohesion	Cohesion is the ability of family members to get along with each other. Categorized into two: ‘Poor’ (coded as 0) and ‘Strong’ (coded as 1).
Ongoing medical conditions	Whether the participant has any of the following ongoing medical conditions: eczema, hay fever, allergies, musculoskeletal problems, ADHD, anxiety, depression, autism, diabetes, asthma, palpitations, congenital heart disease, seizures/epilepsy, wheezing, chronic fatigue, or Disability. The response categories for each condition were ‘Yes/No’. From the responses for each of the categories, we created a new binary variable, termed ‘Any medical conditions’ and coded 1 for ‘Yes’ and 0 for ‘No’.
Psychological distress	Psychological distress was measured using the Kessler Psychological Distress Scale (K10) and categorized based on the K10 scale summed score. For analytical purposes, psychological distress was categorized into three levels: ‘low’ (coded as 0), ‘moderate’ (coded as 1), and ‘high’ (coded as 2)
COVID-19 tested	Whether the respondent tested for COVID-19 or not. The response categories were ‘Yes’ (coded as 1) and ‘No’ (coded as 0). Note that only the Polymerase chain reaction (PCR) testing method was used by the Australian Government until November 2021.
Physical activity during lockdown *	Whether the study participant performed physical activities during the coronavirus restriction period or not. Responses were ‘Yes’ (coded as 1) and ‘No’ (coded as 0).
Employment status in lockdown	The employment status of the respondents during lockdown was dichotomized into two categories: ‘Yes’ (coded as 1) and ‘No’ (coded as 0).
Coronavirus supplement during lockdown	Whether the respondent received any financial support (e.g., Youth Allowance, JobSeeker, or JobKeeper) from the Australian Government during the 1st lockdown due to the COVID-19 pandemic in Australia. Responses were ‘Yes’ (coded as 1) and ‘No’ (coded as 0).
The difficulty of life in lockdown	Addressing the question: How difficult was life during COVID-19 restrictions? Responses included from no problems/stresses to many problems/stresses. The responses were ‘less/no’ (coded as 0) and ‘few/many’ (coded as 1).

* It is the first coronavirus restriction period between March and May 2020 in Australia.

**Table 2:** Sample characteristics (n = 1110).

	n	%
Age ^1^	Mean = 20.63, SD = ±0.49	
Sex		
Male	459	41.4
Female	651	58.6
Country of birth		
Overseas	56	5.0
Australia	1054	95.0
Residential state		
Others	286	25.8
NSW	319	28.7
VIC	298	26.8
QLD	207	18.7
Remoteness		
Major cities	849	76.5
Regional/Remote	261	23.5
Education		
Technical/Others	405	36.5
University/Tertiary	705	63.5
Employment		
Unemployed	248	22.3
Employed	862	77.7
Living with parents		
No	316	28.5
Yes	794	71.5
Family cohesion		
Poor	173	15.6
Strong	937	84.4
IRSAD Quintiles		
Q1 (0–20%)—Most disadvantaged	288	26.0
Q2 (20–40%)	203	18.3
Q3 (40–60%)	268	24.1
Q4 (60–80%)	179	16.1
Q5 (80–100%)—Most advantaged	172	15.5
Ongoing medical conditions		
No	422	38.0
Yes	688	62.0
Psychological distress		
Low	344	31.0
Moderate	308	27.7
High	458	41.3

^1^ Continuous variable—Mean and Standard division presented.

**Table 3:** Reasons for avoiding services among the young people who perceived the need for health services.

Reasons *		COVID-19 Pandemic (n = 440)		Pre-COVID-19 Pandemic (n = 460)	
		n (%)	*p*-Value ***	n (%)	*p*-Value ***
1	Did not know who to go and see	71 (16.1)	<0.001	47 (10.2)	<0.001
2	Had no transportation	18 (4.1)	<0.001	11 (2.4)	0.088
3	No one available to go along with	16 (3.6)	<0.001	11 (2.4)	0.026
4	Difficult to make an appointment	78 (17.7)	<0.001	51 (11.1)	<0.001
5	Afraid of what doctors would say or do	116 (26.4)	<0.001	84 (18.3)	<0.001
6	Thought the problem would go away	246 (55.9)	<0.001	164 (35.7)	<0.001
7	Could not pay	65 (14.8)	<0.001	47 (10.2)	<0.001
8	The problem went away	120 (27.3)	<0.001	82 (17.8)	<0.001
9	Too embarrassed	84 (19.1)	<0.001	59 (12.8)	<0.001
10	Felt I would be discriminated against	10 (2.3)	<0.001	8 (1.7)	0.013
11	Did not think they could help me	78 (17.7)	<0.001	53 (11.5)	<0.001
12	Services not available in my area	14 (3.2)	<0.001	9 (2.0)	0.081
13	Others	65 (14.8)	<0.001	44 (9.6)	<0.001
	During COVID-19 lockdown **		<0.001		
14	I did not want to visit the doctor during the coronavirus restriction period	96 (21.8)	<0.001	-	-
15	My doctor did not perform non-emergency appointments during the coronavirus restriction period	15 (3.4)	<0.001	-	-
16	Appointment cancelled or deferred indefinitely because of the coronavirus restriction period	8 (1.8)	<0.001	-	-
17	Isolating due to the coronavirus restrictions	12 (2.7)	<0.001	-	-
18	A telehealth appointment was the only option available	37 (8.4)	<0.001	-	-

* Reasons are not mutually exclusive, and the respondent had the option not to answer. Here, we only included those who responded ‘Yes’ to the above-mentioned reasons for not accessing services although they perceived the need. ** Coronavirus Restriction Period (CRP) related data not collected in the pre-COVID-19 pandemic period in 2018. *** *p*-value obtained from the two-sample test of proportions, the comparator group, i.e., compared to those who did not avoid health services.

**Table 4:** Factors associated with service access during COVID-19 pandemic (Wave 9C1) and pre-COVID-19 (Wave 8)—Bivariate analysis.

	COVID-19 Pandemic			Pre-COVID-19 Pandemic		
	Service Avoided (n = 440)	Service Accessed (n = 670)	χ^2^ Tests (*p*-Value)	Service Avoided (n = 460)	Service Accessed (n = 650)	χ^2^ Tests (*p*-Value)
Age	Mean = 20.64 (SD = 0.48)	Mean = 20.63 (SD = 0.48)		Mean = 20.63 (SD = 0.48)	Mean = 20.64 (SD = 0.48)	
Sex			8.18 (0.004 **)			4.53 (0.033 *)
Male	159 (34.6)	300 (65.4)		173 (37.7)	286 (62.3)	
Female	281 (43.2)	370 (56.8)		287 (44.1)	364 (55.9)	
Country of birth			0.11 (0.737)			1.37 (0.242)
Overseas	21 (37.5)	35 (62.5)		19 (33.9)	37 (66.1)	
Australia	419 (39.8)	635 (60.2)		441 (41.8)	613 (58.2)	
Residential state			1.15 (0.764)			2.51 (0.472)
Others	118 (41.3)	168 (58.7)		110 (38.5)	176 (61.5)	
NSW	121 (37.9)	198 (62.1)		136 (42.4)	183 (57.4)	
VIC	115 (38.6)	183 (61.4)		132 (44.3)	166 (55.7)	
QLD	86 (41.5)	121 (58.5)		82 (39.6)	125 (60.4)	
Remoteness			0.25 (0.617)			0.55 (0.458)
Major cities	340 (40.1)	509 (59.9)		357 (42.1)	492 (57.9)	
Regional/Remote	100 (38.3)	161 (61.7)		103 (39.5)	158 (60.5)	
Education			0.00 (0.953)			0.61 (0.435)
Technical/Others	161 (39.8)	244 (60.2)		174 (42.9)	231 (57.1)	
University/Tertiary	279 (39.6)	426 (60.4)		286 (40.6)	419 (59.4)	
Employment			1.64 (0.200)			0.03 (0.858)
Unemployed	107 (43.2)	141 (56.9)		104 (41.9)	144 (58.1)	
Employed	333 (38.6)	529 (61.4)		356 (41.3)	506 (58.7)	
Living with parents			3.49 (0.062)			8.85 (0.003 **)
No	139 (44.0)	177 (56.0)		153 (48.4)	163 (51.6)	
Yes	301 (37.9)	493 (62.1)		307 (38.7)	487 (61.3)	
Family cohesion			18.49 (<0.001 ***)			6.62 (0.010 **)
Poor	94 (54.3)	79 (45.7)		87 (50.3)	86 (49.7)	
Strong	346 (36.9)	591 (63.1)		373 (39.8)	564 (60.2)	
IRSAD Quintiles			6.26 (0.180)			5.38 (0.250)
Q1 (0–20%)—Most disadvantaged	112 (38.9)	176 (61.1)		117 (40.6)	171 (59.4)	
Q2 (20–40%)	82 (40.4)	121 (59.6)		81 (39.9)	122 (60.1)	
Q3 (40–60%)	119 (44.4)	149 (55.6)		126 (47.0)	142 (53.0)	
Q4 (60–80%)	71 (39.7)	108 (60.3)		73 (40.8)	106 (59.2)	
Q5 (80–100%)—Most advantaged	56 (32.6)	116 (67.4)		63 (36.6)	109 (63.4)	
Ongoing medical conditions			16.64 (<0.001 ***)			11.38 (0.001 **)
No	135 (32.0)	287 (68.0)		148 (35.1)	274 (64.9)	
Yes	305 (44.3)	383 (55.7)		312 (45.4)	376 (54.7)	
Psychological distress			95.31 (<0.001 ***)			38.59 (<0.001 ***)
Low	75 (21.8)	269 (78.2)		220 (33.7)	432 (66.3)	
Moderate	111 (36.0)	197 (64.0)				
High	254 (55.5)	204 (44.5)		240 (52.4)	218 (47.6)	
COVID-19-tested			0.03 (0.854)			
Yes	129 (40.1)	193 (59.9)		-	-	-
No	311 (39.5)	477 (60.5)				
Physical activity during lockdown			3.79 (0.050 *)			
No	160 (43.7)	206 (56.3)		-	-	-
Yes	280 (37.6)	464 (62.4)				
Employment status during lockdown			2.43 (0.119)			
Unemployed	285 (38.1)	464 (61.9)		-	-	-
Employed	155 (42.9)	206 (57.1)				
Coronavirus supplement during lockdown			1.45 (0.228)			
No	266 (38.3)	429 (61.7)		-	-	-
Yes	174 (41.9)	241 (58.1)				
The difficulty of life during lockdown			24.61 (<0.001 ***)			
Less or no	318 (45.2)	386 (54.8)		-	-	-
Few to many	122 (30.1)	284 (69.9)				

Level of significance considered: * *p* < 0.05, ** *p* < 0.01, *** *p* < 0.001.

**Table 5:** Determinants of service avoidance among young people who perceived the need for healthcare (COVID-19 vs. pre-COVID-19).

	Model I (COVID-19 Pandemic) aOR (95% CI) ^1^	Model II (Pre-COVID-19) aOR (95% CI)
Sex		
Male	Ref.	Ref.
Female	1.27 * (1.01, 1.65)	1.11 (0.94, 1.32)
Living with parents		
No	Ref.	Ref.
Yes	0.93 (0.67, 1.31)	0.73 (0.46, 1.16)
Family cohesion		
Poor	Ref.	Ref.
Strong	0.73 (0.51, 1.10)	0.70 (0.38, 1.29)
Ongoing medical conditions		
No	Ref.	Ref.
Yes	1.38 * (1.13, 1.70)	1.33 (0.91, 1.95)
Psychological distress		
Low	Ref.	Ref.
Moderate	2.06 ** (1.35, 3.18)	1.72 *** (1.31, 2.26)
High	4.77 *** (3.57, 6.37)	2.97 *** (2.11, 4.16)
Physical activity during lockdown		
No	Ref.	-
Yes	0.85 (0.63, 1.16)	
Difficulties of life in lockdown		
Less difficulty or no	Ref.	-
Few to many	0.81 (0.65, 1.02)	

^1^ Adjusted odds ratio (OR) with 95% confidence interval (CI). Level of significance considered: * *p* < 0.05, ** *p* < 0.01, *** *p* < 0.001.

## Data Availability

The LSAC datasets are available through the National Centre for Longitudinal Data (NCLD) Dataverse, which is publicly accessible upon request, available at https://growingupinaustralia.gov.au/data-and-documentation/accessing-lsac-data (accessed on 15 July 2021). It is not permissible for authors to share the unit record data without approval from the Department of Social Services (DSS) and the Australian Institute of Family Studies (AIFS).
